# Focus Where It Matters: Design and Implementation of a Physiology‐Based Care Pathway for Traumatic Shock

**DOI:** 10.1111/1742-6723.70391

**Published:** 2026-11-04

**Authors:** Michael Noonan, Joseph Mathew, Simon Hendel, Andrew Underhill, Jean‐Philippe Miller, Alexa Cass, Hector Thomson, Holly Bannon‐Murphy, Mark Fitzgerald

**Affiliations:** ^1^ National Trauma Research Institute Melbourne Australia; ^2^ Trauma Service, The Alfred Hospital Melbourne Australia; ^3^ Emergency & Trauma Centre, The Alfred Hospital Melbourne Australia; ^4^ School of Translational Medicine, Monash University Australia; ^5^ Department of Anaesthesiology and Perioperative Medicine The Alfred Hospital Melbourne Australia

**Keywords:** clinical pathway, polytrauma, quality improvement, resuscitation, traumatic shock

## Abstract

**Objective:**

Traumatic shock lacks a consensus definition, and in blunt‐predominant systems its causes are heterogeneous. Simple physiological parameters remain the most universally available basis for early recognition. We describe the design and staged implementation of a physiology‐based care pathway for traumatic shock and evaluate its implementation through staff experience.

**Methods:**

Single‐centre quality‐improvement and implementation project at an Australian Level 1 trauma centre, reported per SQUIRE 2.0. The pathway comprised physiology‐based activation (systolic blood pressure ≤ 90 mmHg, shock index > 1, prehospital blood or senior clinician concern), structured pre‐arrival preparation, trauma bay and equipment standardisation, task‐based team roles, prioritised life‐saving interventions and senior‐led disposition, supported by simulation and embedded quality improvement. Components were implemented between June and November 2022. Evaluation comprised pre‐intervention (2021) and post‐implementation (2025) staff surveys, summarised as top‐box favourability, with cross‐sectional characterisation of the shocked cohort. Clinical effectiveness was not assessed.

**Results:**

Of 3667 major trauma patients, 324 (8.8%) met shocked trauma criteria; these patients were younger, more severely injured and had markedly higher mortality (emergency department death 6.8% vs. 0.4%). Favourable ratings of trauma bay equipment and environment rose from approximately one‐quarter to 87.0%. Post‐implementation, staff reported high favourability for timely life‐saving interventions (88.9%), pathway embedding (85.2%) and role confidence (86.5%). Leadership clarity, communication and reliable debriefing did not improve and were identified as ongoing targets.

**Conclusions:**

A physiology‐based traumatic shock pathway was feasibly implemented and embedded, with high staff‐reported readiness and clear targets for refinement. Effectiveness remains to be established.

## 1 Introduction

Injury causes approximately 4.4 million deaths worldwide each year and remains the leading cause of death among Australians aged 1–44 years [1, 2]. A substantial proportion of these deaths occur within hours of injury, with traumatic shock implicated in many of them [3].

Shock is a state of profound and widespread reduction in effective tissue perfusion that, if not promptly reversed, progresses from cellular injury to organ failure and death [4]. In the injured patient this state is not the product of a single mechanism. Haemorrhage is the dominant and most time‐critical contributor, but tension physiology, cardiac tamponade, neurogenic injury and direct myocardial insult may each produce or compound inadequate perfusion, and these mechanisms frequently coexist and evolve during the first minutes of care [4, 5]. Traumatic shock is therefore better understood as a heterogeneous syndrome than as a single disease entity; a distinction with direct consequences for how it is recognised and treated, yet one for which no consensus clinical or research definition exists [6].

Such heterogeneity is greatest in blunt‐predominant systems, which characterise Australian and comparable trauma populations. In a recent analysis of our institutional major trauma cohort, haemorrhage accounted for approximately two‐thirds of primary aetiologies of traumatic shock; the remaining third were attributable to non‐haemorrhagic, indeterminate or mixed mechanisms, two or more contributing causes were identified in 44.0% of cases, and indeterminate aetiology was more frequent following blunt than penetrating injury [7]. These findings indicate that early physiological compromise in this population cannot be assumed to be haemorrhagic in origin, and that a diagnostic approach centred exclusively on haemorrhage risks misdirecting resuscitation in a substantial minority of patients, favouring a recognition strategy that is agnostic to the underlying mechanism.

In the absence of an agreed definition, that recognition rests on simple physiological parameters. Low arterial pressure has signalled poor survival since the first systematic battlefield observations [8], and the shock index was introduced to improve on pulse rate and blood pressure considered separately [9]. Prehospital hypotension remains a valid trigger for trauma team activation [10]. Systolic blood pressure (SBP) and shock index are imperfect, but they are universally available, reproducible and interpretable within seconds, and they identify a subgroup with substantially higher mortality and resource requirements; in a national registry analysis currently under review, we observed a stepwise rise in mortality across the range of thresholds historically used to define hypotension. These parameters therefore offer a pragmatic operational basis for identifying patients who warrant an escalated, standardised response.

Delivering that response reliably is itself difficult. Early trauma reception is a period of exceptional cognitive load, in which a critical decision linked to a life‐saving intervention is made approximately every 72 s during the first 30 min [11], and errors of omission remain common even in mature trauma centres. Human‐factors evidence indicates that structured workflows, explicit role allocation, standardised equipment and team time‐out processes reduce such error and support the reliable delivery of time‐critical care [11, 12]. For a physiologically unstable and mechanistically heterogeneous population, in which the dominant cause of shock may not be immediately apparent, a standardised and senior‐led reception process offers a means of ensuring that essential interventions are neither delayed nor missed.

We therefore developed the Shocked Trauma Care Pathway, which links a pragmatic, physiological trigger to a structured, senior‐led resuscitation response. The pathway combines standardised activation criteria, structured pre‐arrival preparation, trauma bay and equipment standardisation, task‐based team roles, prioritised life‐saving interventions and rapid disposition, supported by simulation and an embedded quality‐improvement programme. This paper describes the design and staged implementation of the pathway and evaluates its implementation through staff experience and cross‐sectional characterisation of the shocked trauma cohort. Clinical effectiveness was not assessed.

## 2 Methods

### 2.1 Study Design and Reporting

We report the design, staged implementation and implementation evaluation of a care pathway for trauma patients with early clinical signs of shock, conducted as a single‐centre quality‐improvement and implementation project and reported in accordance with the SQUIRE 2.0 guidelines [13]. The improvement aims of the pathway were to standardise reception and resuscitation, reduce errors of omission, facilitate effective team dynamics, and reduce time to life‐saving interventions (LSIs) and to time‐critical surgical or interventional radiology procedures. The aims of the present evaluation were narrower: to describe the intervention and its staged implementation, to assess adoption and staff experience, and to characterise the shocked trauma cohort cross‐sectionally. Clinical effectiveness was not evaluated, as no pre‐implementation patient cohort with resuscitation‐phase process data existed; the shocked trauma call‐out and the prospective resuscitation registry both post‐date the intervention.

### 2.2 Setting

The project was conducted at The Alfred, Melbourne, an adult Level 1 trauma centre with 24‐h in‐house emergency medicine, anaesthesia, intensive care, radiology and trauma surgery. The centre is one of two adult Major Trauma Services in Victoria, manages approximately half of the state's major trauma admissions [14], and is among the highest‐volume contributors to the Australia New Zealand Trauma Registry [15].

### 2.3 Intervention Development and Components

The pathway was developed from 2019 by a multidisciplinary working group comprising emergency medicine, trauma surgery, anaesthesia, intensive care, radiology, trauma nursing and prehospital stakeholders, informed by registry and human‐factors literature, local epidemiology and observational review of trauma reception. Design drivers included the heterogeneity of shock presentations in a blunt‐predominant population, the absence of an agreed definition of traumatic shock, observed variability in early resuscitation, and the cognitive load and error risk of trauma reception.

The pathway comprises 10 components (Table 1, Figures 2 and 3) implemented in stages between June and November 2022, with the shocked trauma call‐out going live on 22 November 2022 (Figure 1). Activation required standard trauma call‐out criteria plus at least one of: SBP 90 mmHg or less; shock index (heart rate divided by SBP) greater than 1; administration of any prehospital blood product; or senior clinician concern regarding early traumatic shock physiology. Prehospital blood administration in our system is governed by the same physiological thresholds (SBP ≤ 90 mmHg or shock index > 1). This criterion was therefore included to capture patients who met these thresholds in the field and received prehospital blood, but who had been resuscitated above them by the time of arrival; without it, physiologically shocked patients successfully treated before arrival would be systematically excluded.

**TABLE 1:** Shocked Trauma Care Pathway—intervention components.

Component (date implemented)	Description	Intended mechanism/evidence base
Shocked‐trauma activation criteria and call‐out (Nov 2022)	Physiology‐based trigger appended to the standard trauma page: SBP ≤ 90 mmHg, shock index > 1, any prehospital blood product or senior‐clinician concern. Announced as ‘SHOCKED TRAUMA’.	Scales the most resource‐intensive response to the highest‐risk minority. Prehospital blood criterion included to capture patients resuscitated above SBP/SI thresholds before arrival [10, 16].
Reception and resuscitation guideline plus supplementary clinician guideline (Jun 2022)	Standardised pre‐arrival, reception, LSI‐prioritisation and senior‐led disposition workflow, built on the Trauma Team Time‐Out framework.	Reduces errors of omission and cognitive load in time‐critical care [12, 17, 18].
Shocked‐trauma reception checklist (Jun 2022; current version [v11—confirm])	Four‐phase cognitive aid (Team—Equipment—Plan—Patient arrival) supporting pre‐arrival preparation and pre‐ and post‐arrival time‐outs.	Cognitive offloading and reduced omissions via structured prompts [12, 13, 18].
Standardised trauma bay layout and equipment guideline (Jun 2022; bays 2–3 renovated Jul 2022)	Fixed patient positioning, defined team zones, standardised trolleys and packs, equipment scouts, and cordoned set‐up and observer areas.	Reduces environmental distraction and economises team‐member movement [13].
Mandatory senior notification and defined roles (Jun and Nov 2022)	Required core‐member attendance (emergency, trauma, anaesthesia, intensive care, radiographer, ward support) with explicit role and zone allocation.	Senior‐led decision‐making; clear leadership and followership [11, 17].
Daily trauma team huddles (Jun 2022)	Standardised briefings at 0900, 1700 and 2300 for role allocation and LSI planning.	Shared mental model and team familiarity.
Multidisciplinary in situ simulation (from Oct 2022)	Translational simulation used to iterate and refine the pathway, followed by recurrent monthly multidisciplinary in situ simulation incorporating learnings from ATRQI case review.	Familiarity with equipment, roles and workflow; iterative refinement of the pathway.
Trauma QI reporting form and ATRQI working group (Oct and Nov 2022; revised May and Nov 2023)	Structured quality capture with monthly multidisciplinary case discussion and aggregate data review by the Alfred Trauma Resuscitation Quality Improvement (ATRQI) working group.	Continuous improvement feedback loop driving iterative pathway refinement.
Structured case‐outcome feedback (from 2022 to 2023)	Case‐outcome feedback to attending staff and multidisciplinary case reviews.	Staff closure, education and engagement.
Alfred Trauma Resuscitation Registry (formalised Nov 2023)	Prospective capture of resuscitation‐phase process data.	Enables audit and prospective evaluation of the pathway.

Abbreviations: ATRQI, Alfred Trauma Resuscitation Quality Improvement working group; LSI, life‐saving intervention; SBP, systolic blood pressure; SI, shock index.

**FIGURE 1:**
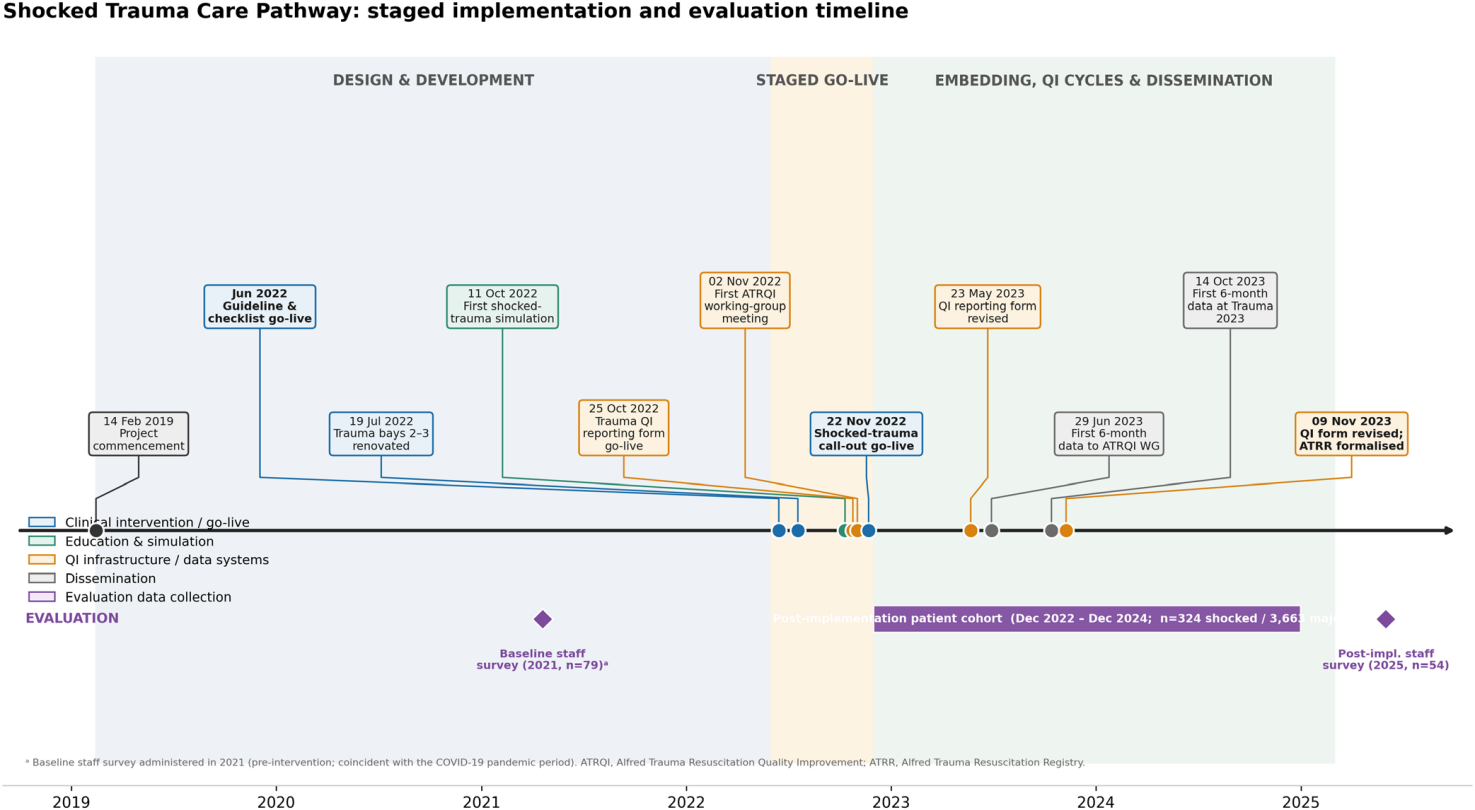
Staged implementation and evaluation timeline for the Shocked Trauma Care Pathway. Intervention components (Table 1) were introduced progressively between June and November 2022; the shocked trauma call‐out went live on 22 November 2022. Evaluation comprised a pre‐intervention staff survey (2021), a post‐implementation staff survey (2025) and a post‐implementation patient cohort (1 December 2022 to 30 January 2025). The baseline staff survey coincided with the COVID‐19 pandemic period. ATRQI, Alfred Trauma Resuscitation Quality Improvement working group; ATRR, Alfred Trauma Resuscitation Registry; LSI, life‐saving intervention.

Activation triggered mandatory senior notification, standardised trauma bay preparation (Figure 2) and allocation of predefined task‐based team roles. Reception followed a structured checklist across four phases: team, equipment, plan and patient arrival (Figure 3), built on the Trauma Team Time‐Out framework [17]. Implementation was supported by multidisciplinary translational simulation during development and, from October 2022, recurrent monthly in situ simulation. Governance was provided by the Alfred Trauma Resuscitation Quality Improvement (ATRQI) working group, established in November 2022, which undertakes monthly multidisciplinary case discussion and aggregate data review and returns learnings to the clinical floor through simulation and guideline revision. Structured data collection commenced with a trauma quality‐improvement reporting form in October 2022 and was formalised as the Alfred Trauma Resuscitation Registry in November 2023. The full workflow, team roles, equipment specifications and quality‐improvement framework are provided in Appendix S1, with the standardised bay illustrated in Figure S1.

**FIGURE 2:**
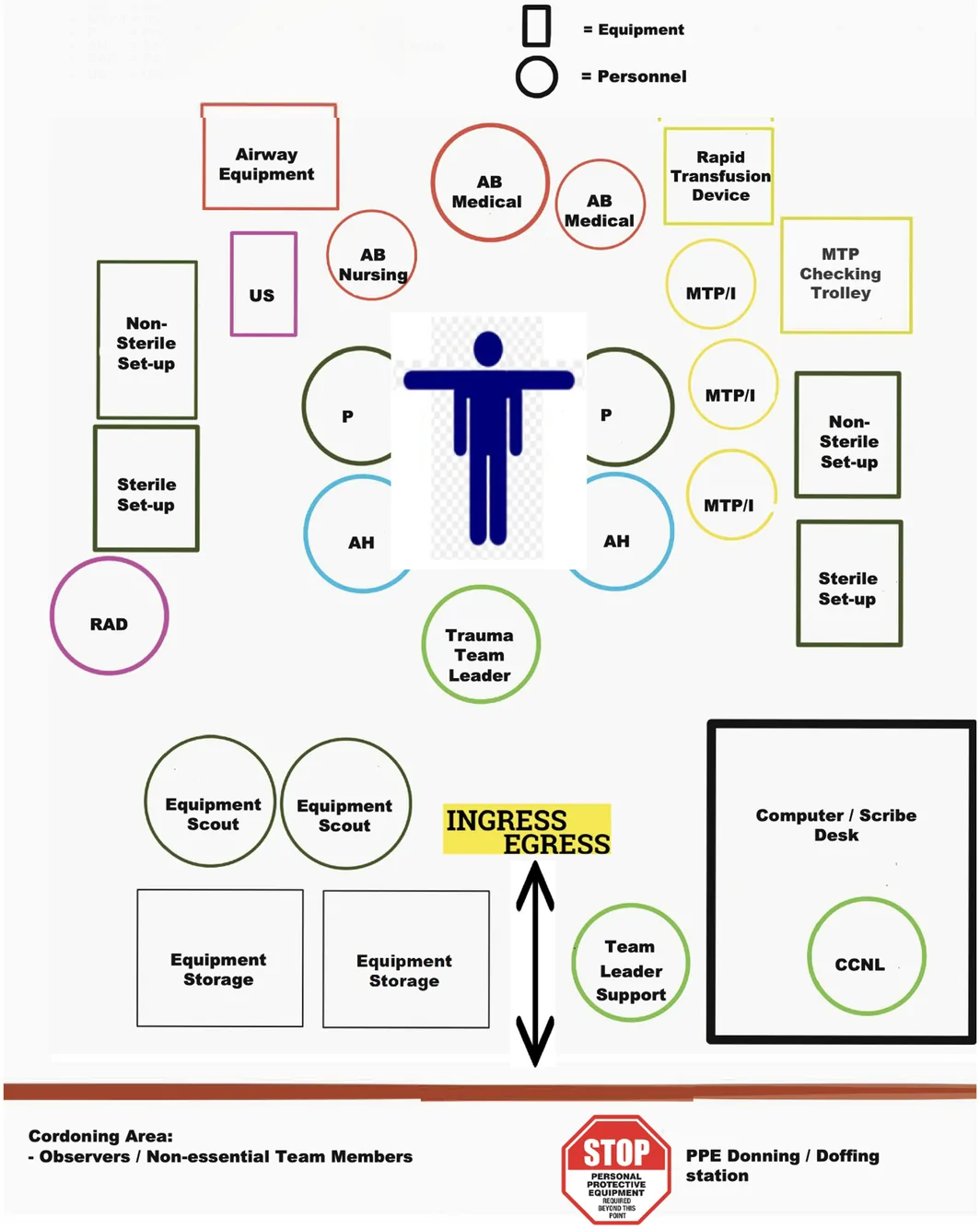
Standardised trauma bay set‐up for shocked trauma reception (schematic). AB, airway and breathing team; AH, assessment and haemorrhage control team; MTP/I, massive transfusion and infusion team; P, procedure team; RAD, radiographer; US, ultrasound.

**FIGURE 3:**
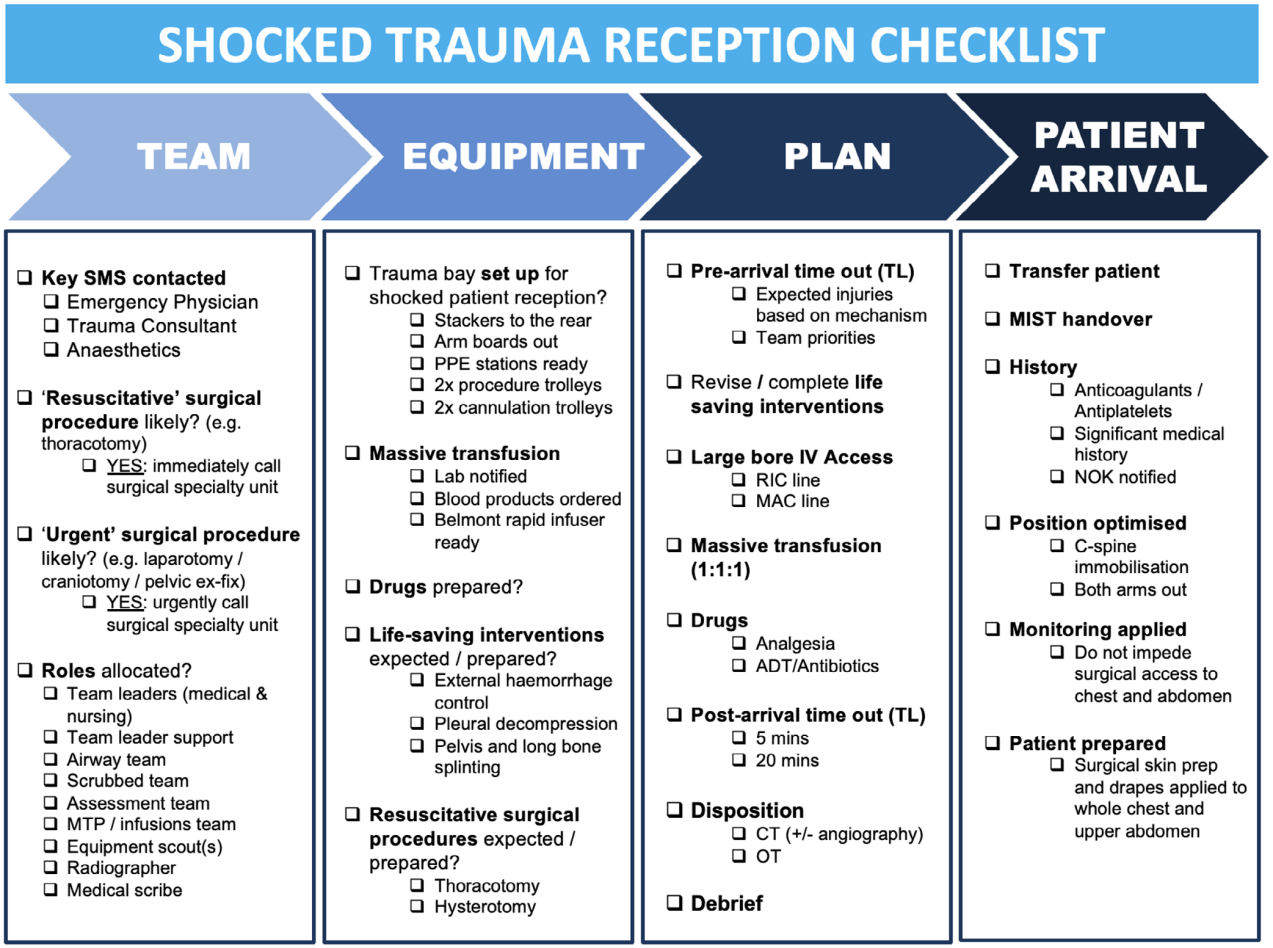
Shocked trauma reception checklist.

### 2.4 Evaluation: Staff Experience

Two cross‐sectional anonymous electronic surveys of the multidisciplinary trauma workforce were undertaken. A pre‐intervention baseline survey was administered in 2021 (79 respondents), before any pathway component went live, rating trauma bay, equipment, team and debriefing domains on a five‐point quality scale (*Very Poor* to *Excellent*). A post‐implementation survey was administered in 2025 (54 respondents), evaluating experience over the preceding 3 months across trauma bay environment, team structure and communication, timeliness and workflow, pathway embedding, education and debriefing, on a five‐point agreement scale (*Strongly Disagree* to *Strongly Agree*). The instruments were designed to address equivalent domains but were not identical: The 2021 survey was administered during the COVID‐19 pandemic, when departmental priorities, infection‐control requirements and personal protective equipment use differed materially, and it accordingly included items with no post‐implementation counterpart. Both instruments are provided in Appendices S2 and S3. Completion implied consent and responses were analysed in aggregate.

Because the instruments differed in wording and response scale and sampled independent, non‐paired convenience samples separated by approximately 4 years, responses were summarised descriptively as the proportion selecting the two most favourable categories (‘top‐box’ favourability); no inferential comparison was performed. Responses of ‘not applicable’ were excluded from item denominators. Free‐text responses were reviewed to identify recurrent barriers to timely care.

### 2.5 Evaluation: Patient Cohort

All adult major trauma patients (aged 16 years or older, Injury Severity Score 13 or greater) presenting between 1 December 2022 and 30 January 2025 were identified from the Alfred Health Trauma Registry. Patients meeting the shock criteria above were characterised descriptively and compared with those not meeting criteria. Detailed aetiology, resource utilisation and operative burden for this cohort are reported separately [7, 18].

### 2.6 Analysis

Categorical variables are reported as counts and percentages, and continuous variables as means with standard deviations or medians with interquartile ranges. Survey items are reported as top‐box favourability. No inferential testing was performed.

### 2.7 Ethics

The staff experience surveys were conducted as a research project approved by the Bayside Health Human Research Ethics Committee (Project No. 811/20); participation was voluntary, responses were anonymous and analysed in aggregate, and completion of the survey implied consent to participate. Patient cohort analyses used registry data collected and analysed under institutional quality‐improvement governance. No patient‐identifiable data are reported.

## 3 Results

### 3.1 Patient Cohort

Between 1 December 2022 and 30 January 2025, 3667 adult major trauma patients presented to the trauma centre, of whom 324 (8.8%) met shocked trauma criteria. Patients meeting criteria were younger than those who did not (mean 46.4 vs. 57.8 years), more severely injured (median ISS 29, IQR 20–41, vs. 17, IQR 13–22), and more frequently transported by helicopter (42.3% vs. 12.5%). Blunt injury predominated in both groups (86.7% vs. 94.0%), with penetrating mechanisms over‐represented among shocked patients (13.0% vs. 2.2%). Mortality was substantially higher in the shocked cohort, both in the emergency department (6.8% vs. 0.4%) and during the acute admission (22.5% vs. 7.8%). Detailed aetiology, resource utilisation and operative burden for this cohort are reported separately [7, 18].

### 3.2 Staff Experience

The baseline survey was completed by 79 staff in 2021 and the post‐implementation survey by 54 staff in 2025, the latter including a higher proportion of senior medical and multidisciplinary respondents (Table S1). Top‐box favourability by domain is shown in Table 2. Because the two surveys used different instruments and independent samples, both increases and decreases in favourability are interpreted descriptively and cautiously and should not be read as measured change.

**TABLE 2:** Staff experience before and after implementation (top‐box favourability).

Domain/item	Pre‐implementation 2021, % favourable^a^ (*n* = 79)	Post‐implementation 2025, % favourable^a^ (*n* = 54)
Trauma bay environment and equipment		
Equipment readily available and appropriate	26.6	87.0
Bay layout and set‐up predictable and supports movement^b^	29.1	87.0
Positioning allows adequate procedural access	41.8	79.6
Team structure, leadership and communication		
Senior specialty clinicians attend when required	88.6	81.5
Pre‐arrival huddle or briefing occurs	69.6	79.6
Team roles clearly allocated	83.5	79.6
Leadership clear and consistent	81.0	59.3
Communication between team members effective	83.5	64.8
Followership and task focus	81.0	74.1
Timeliness and workflow		
Life‐saving interventions delivered in a timely manner	—	88.9
Disposition decisions (CT vs. OT or IR) clear	—	70.4
Transfer to definitive care (OT or IR) without delay	—	59.3
Pathway embedding and culture		
Pathway embedded in routine practice	—	85.2
Pathway promotes shared understanding of priorities	—	83.3
Pathway increases confidence in role (*n* = 52)	—	86.5
Education and training		
Education accessible and relevant (*n* = 51)	—	70.6
In situ simulation improved familiarity (*n* = 45)	—	68.9
Debriefing and staff support		
Colleague support during resuscitation	83.5	63.0
Colleague support after resuscitation	68.4	61.1
Formal post‐case debrief occurs reliably	49.4	40.7
Follow‐up after debriefing appropriate	11.4	51.9
Case‐outcome feedback (introduced with pathway)		
Follow‐up outcome emails useful (*n* = 45)	—	57.8
Case‐outcome information aided education (*n* = 45)	—	66.7
Case‐outcome information aided closure (*n* = 45)	—	62.2
Overall		
Overall resuscitation well organised and consistent	89.9^c^	87.0

^a^ % Favourable = proportion selecting the two most favourable categories (*Good* or *Excellent* pre‐implementation; *Agree* or *Strongly Agree* post‐implementation). The pre‐implementation instrument used a five‐point quality scale (*Very Poor* to *Excellent*) and the post‐implementation instrument a five‐point agreement scale (*Strongly Disagree* to *Strongly Agree*). Instruments differed in part because the 2021 baseline was administered during the COVID‐19 pandemic, when departmental priorities and infection‐control requirements differed. The surveys were independent, non‐paired cross‐sectional convenience samples separated by approximately 4 years; the comparison is indicative only and no inferential testing was undertaken. ‘Not applicable’ responses were excluded from denominators, giving reduced n where indicated. Respondent characteristics are provided in Table S1.

^b^ Pre‐implementation value is ‘ease of set‐up for shocked trauma patients’; the companion item ‘ease of team‐member movement to access equipment’ was rated 34.2% favourable.

^c^ Pre‐implementation value is the nearest conceptual item (‘overall team function’); wording differs from the post‐implementation item.

The largest shift was in the trauma bay environment. Favourable ratings of equipment availability and appropriateness rose from approximately one‐quarter of respondents at baseline to 87.0%, with comparable gains in perceived layout consistency (29.1%–87.0%) and procedural access (41.8%–79.6%). This is the domain most directly targeted by the bay standardisation and equipment components of the pathway; however, because the 2021 baseline was administered during the COVID‐19 pandemic, when infection‐control requirements and personal protective equipment use materially altered the resuscitation environment, this change reflects the combined effect of physical standardisation and the resolution of pandemic‐era constraints rather than the pathway alone.

Team‐level domains were rated favourably at baseline and did not improve. Favourability for leadership clarity (59.3%) and inter‐team communication (64.8%) was lower post‐implementation, and free‐text responses identified duplicated or unclear medical leadership between trauma and emergency streams, crowding of the resuscitation bay by non‐essential personnel, and non‐streamlined interventional radiology access as persistent barriers. Given the different response scale and the more senior, more critical composition of the later sample, these values are interpreted, like the favourable domains, as indicative rather than measured, and identify active targets for refinement.

Post‐implementation, staff reported high favourability for timely delivery of LSIs (88.9%), pathway embedding in routine practice (85.2%), shared understanding of clinical priorities (83.3%) and role‐related confidence (86.5%); overall, 87.0% agreed that shocked trauma resuscitation was well organised and consistent. Within the workflow domain, transfer to definitive care without unnecessary delay was the weakest item (59.3% favourable, 20.4% unfavourable), consistent with the interventional radiology access barriers raised in free text.

Debriefing and structured feedback showed a mixed picture. Reliable formal post‐case debriefing remained an area for development (40.7%) and was largely unchanged from baseline (49.4%). In contrast, structured case‐outcome feedback introduced with the pathway was valued (57.8%–66.7% across items), contrasting with historically low satisfaction with post‐case follow‐up at baseline (11.4%). Recurrent free‐text requests included clearer senior medical role allocation, attendance and crowd‐control measures, point‐of‐care coagulation testing and expanded pharmacy involvement in simulation. No pathway‐related safety signals were identified through routine ATRQI review.

## 4 Discussion

We describe the design and staged implementation of a physiology‐based care pathway for traumatic shock in an Australian Level 1 trauma centre. Implementation was feasible, the pathway became embedded as the default approach to shock physiology, and staff‐reported readiness of the resuscitation environment improved markedly. The evaluation also identified specific and actionable deficits in leadership clarity, resuscitation bay crowd control, interventional radiology access and reliable debriefing.

The pattern of findings is consistent with the mechanism by which the pathway was designed to act. Components directly targeting the physical environment, namely standardised bay layout, equipment guidelines and checklist‐driven preparation, corresponded to the largest gains in staff‐reported readiness, in keeping with human‐factors evidence that environmental standardisation reduces cognitive load and supports reliable performance under time pressure [11, 12]. Domains dependent on interprofessional behaviour proved more resistant, a common finding in complex team‐based interventions, and one that has redirected local improvement effort toward explicit senior role allocation, attendance control and strengthened debrief structures. The concurrent COVID‐19 context, discussed further below, limits how much of the environmental gain can be attributed to the pathway itself.

The cohort data support concentrating preparation and senior resources on this group. Patients meeting shocked trauma criteria were a small minority of major trauma presentations but were younger, more severely injured, more often transported by helicopter, and had emergency department mortality more than tenfold that of other major trauma patients. Although blunt mechanisms predominated overall, penetrating injuries were over‐represented among shocked patients relative to the wider major trauma population (13.0% vs. 2.2%), consistent with the greater propensity of penetrating trauma to produce early haemodynamic compromise; the pathway's physiological trigger identifies both groups without reliance on mechanism. Their causes of shock are heterogeneous, with approximately one‐third non‐haemorrhagic, indeterminate or mixed and multiple contributory causes common after blunt injury [7], and their resource requirements are disproportionate, frequently multispecialty, iterative and delivered out of hours [18]. This combination of high risk, diagnostic uncertainty and resource intensity is precisely what a standardised, senior‐led response is suited to address, and it argues against relying on a purely haemorrhage‐centred model of early care in blunt‐predominant systems.

Several features support wider applicability. The activation criteria are simple, universally measurable and compatible with existing prehospital and emergency department call‐out systems, which matters in a regionalised system where exposure to major trauma and confidence in trauma team leadership vary considerably between centres [16]. The pathway depends on team structure, communication discipline and human‐factors principles rather than new infrastructure, and its educational components, in situ simulation and daily huddles, are scalable and low cost. Trauma bay standardisation can be adapted using existing equipment inventories. The core principles of structured physiological activation, task‐based senior‐led team processes, standardised environment, simulation and continuous audit are transferable to other Australian and New Zealand services.

The principal unanswered question is clinical effectiveness. This project was conceived from 2019 with a planned pre‐ and post‐implementation comparison of patient‐level process and outcome measures. The onset of the COVID‐19 pandemic interrupted this design: Over the subsequent period, our centre implemented substantial and repeated changes to trauma workflows, personal protective equipment and staffing models, alongside altered patterns of injury presentation, such that the pre‐pandemic period could not serve as a valid comparator for care delivered under the pathway. Structured, resuscitation‐phase process data collection was itself established only as part of the pathway and did not exist beforehand. For these reasons the present evaluation was confined to implementation and staff‐experience measures. The registry infrastructure established by the pathway now permits prospective monitoring of time to computed tomography, transfusion practice and disposition reliability, and provides a platform for evaluating whether standardised early shock care improves patient‐centred outcomes. Multicentre collaboration would strengthen such work and support a shared Australasian model.

## 5 Limitations

As described, the planned before‐and‐after comparison was precluded by pandemic‐era disruption and the absence of pre‐pathway process data; the evaluation is therefore descriptive, and the bundled components cannot be separately attributed. The staff surveys were independent, non‐paired samples 4 years apart using different scales, compared descriptively only; composition differed, response rates were unquantifiable, and the smaller, more senior later sample may rate more critically. The 2021 baseline coincided with COVID‐19, confounding the trauma bay domain in particular. Self‐reported experience proxies performance, and free‐text was reviewed informally. This is a single‐centre evaluation of an evolving initiative.

## 6 Conclusions

A structured, senior‐led, physiology‐based care pathway for traumatic shock was feasibly implemented and embedded into routine practice at an Australian Level 1 trauma centre. Staff‐reported readiness of the resuscitation environment improved markedly, and post‐implementation confidence in role, shared understanding of clinical priorities and perceived organisation of care were high. Leadership clarity, resuscitation bay crowd control, interventional radiology access and reliable debriefing were identified as ongoing targets for refinement. In a blunt‐predominant system where traumatic shock is heterogeneous in cause and disproportionate in resource demand, simple physiological criteria offer a pragmatic basis for identifying patients who warrant an escalated, standardised response. The principles applied here are potentially adaptable to other Australian and New Zealand trauma services, with local tailoring to resource and staffing context. Whether this approach improves patient‐centred outcomes remains to be established and is the focus of ongoing registry‐based evaluation.

## Author Contributions

**Michael Noonan:** conceptualisation, methodology, formal analysis, investigation, data curation, writing – original draft, writing – review and editing, visualisation. **Joseph Mathew:** conceptualisation, writing – review and editing. **Simon Hendel:** conceptualisation, writing – review and editing. **Andrew Underhill:** conceptualisation, writing – review and editing. **Jean‐Philippe Miller:** investigation, data curation, writing – review and editing. **Alexa Cass:** investigation, data curation, writing – review and editing. **Hector Thomson:** writing – review and editing. **Holly Bannon‐Murphy:** investigation, data curation, writing – review and editing. **Mark Fitzgerald:** conceptualisation, writing – review and editing, visualisation, supervision.

## Funding

The authors have nothing to report.

## Conflicts of Interest

The authors declare no conflicts of interest.

## Supporting information


**Table S1:** Staff survey respondent characteristics.
**Figure S1:** Standardised trauma bay set‐up: (a) equipment stackers; (b) prepared trauma bay.
**Appendix S1:** Shocked Trauma Care Pathway: Detailed workflow, team roles, equipment standardisation, implementation strategy and internal quality‐improvement framework.
**Appendix S2:** Pre‐implementation staff survey instrument (2021).
**Appendix S3:** Post‐implementation staff experience survey instrument (2025).

## Data Availability

Data sharing not applicable to this article as no datasets were generated or analyzed during the current study.

## References

[1] World Health Organization , Injuries and Violence: The Facts 2021 (World Health Organization, 2021).

[2] Australian Institute of Health and Welfare , Injury in Australia (AIHW, 2023).

[3] D. S. Kauvar , R. Lefering , and C. E. Wade , “Impact of Hemorrhage on Trauma Outcome: An Overview of Epidemiology, Clinical Presentations, and Therapeutic Considerations,” Journal of Trauma 60, no. 6 Suppl (2006): S3–S11, 10.1097/01.ta.0000199961.02677.19. 16763478

[4] R. A. Manji , K. E. Wood , and A. Kumar , “The History and Evolution of Circulatory Shock,” Critical Care Clinics 25 (2009): 1–29, 10.1016/j.ccc.2008.12.013. 19268792

[5] Z. J. Balogh , “Polytrauma: Acute Acquired Mitochondrial Disease,” Injury 54 (2023): 1407–1408, 10.1016/j.injury.2023.05.019. 37160406

[6] M. Noonan , C. Johnny , Y. Kim , et al., “Methods of Defining Major Haemorrhage After Injury: A Scoping Review,” Health Sciences Review 11 (2024): 100164.

[7] M. Noonan , F. Francis‐Pester , G. O'Reilly , et al., “Aetiology of Traumatic Shock in an Australian Level 1 Trauma Centre,” Emergency Medicine Australasia 38 (2026): e70286, 10.1111/1742-6723.70286. 42203493

[8] W. B. Cannon , J. Fraser , and E. M. Cowell , “The Preventive Treatment of Wound Shock,” JAMA 70 (1918): 618–621.

[9] M. Allgöwer and C. Burri , “Schockindex,” Deutsche Medizinische Wochenschrift 92 (1967): 1947–1950, 10.1055/s-0028-1106070. 5299769

[10] G. A. Franklin , P. W. Boaz , D. A. Spain , J. K. Lukan , E. H. Carrillo , and J. D. Richardson , “Prehospital Hypotension as a Valid Indicator of Trauma Team Activation,” Journal of Trauma 48 (2000): 1034–1037, 10.1097/00005373-200006000-00006. 10866247

[11] M. Fitzgerald , P. Cameron , C. Mackenzie , et al., “Trauma Resuscitation Errors and Computer‐Assisted Decision Support,” Archives of Surgery 146 (2011): 218–225, 10.1001/archsurg.2010.333. 21339436

[12] C. Hicks and A. Petrosoniak , “The Human Factor: Optimizing Trauma Team Performance in Dynamic Clinical Environments,” Emergency Medicine Clinics of North America 36 (2018): 1–17. 10.1016/j.emc.2017.08.003 29132571

[13] G. Ogrinc , L. Davies , D. Goodman , P. Batalden , F. Davidoff , and D. Stevens , “SQUIRE 2.0 (Standards for QUality Improvement Reporting Excellence): Revised Publication Guidelines From a Detailed Consensus Process,” BMJ Quality and Safety 25 (2016): 986–992, 10.1136/bmjqs-2015-004411. PMC5256233 26369893

[14] Victorian State Trauma Outcomes Registry and Monitoring Group , Victorian State Trauma Registry Annual Report 2022–23 (Monash University, 2024).

[15] Australia New Zealand Trauma Registry , Annual Report 2023–24 (National Trauma Research Institute, 2024).

[16] M. Putland , M. Noonan , A. Olaussen , P. Cameron , and M. Fitzgerald , “Low Major Trauma Confidence Among Emergency Physicians Working Outside Major Trauma Services: Inevitable Result of a Centralised Trauma System or Evidence for Change?,” Emergency Medicine Australasia 30 (2018): 834–842, 10.1111/1742-6723.13135. 30054972

[17] M. Fitzgerald , S. Reilly , D. V. Smit , et al., “The World Health Organization Trauma Checklist Versus Trauma Team Time‐Out: A Perspective,” Emergency Medicine Australasia 31 (2019): 882–885, 10.1111/1742-6723.13306. PMC6851662 31081585

[18] M. Noonan , M. O'Loan , S. Hendel , et al., “Operative Burden and Resuscitation Resource Utilisation in Patients With Traumatic Shock Requiring Urgent Surgical or Endovascular Intervention,” European Journal of Trauma and Emergency Surgery 52 (2026): 149. 10.1007/s00068-026-03197-2 PMC13121394 42043478

