# Supplementary material for: Focus Where It Matters: Design and Implementation of a Physiology‐Based Care Pathway for Traumatic Shock
Source: Emerg Med Australas. 2026 Nov 4;38(6):e70391. doi: 10.1111/1742-6723.70391 (PMC13735533; doi:10.1111/1742-6723.70391)
Supplement: Supplementary file 1 [file EMM-38-0-s001.docx]

**Supplementary Material**

Table S1. Staff survey respondent characteristics.

| **Characteristic** | **Pre-implementation 2021 (n=79)** | **Post-implementation 2025 (n=54)** |
| --- | --- | --- |
| **Age (years)** |  |  |
| 20-30 | 21 | 8 |
| 31-40 | 43 | 32 |
| 41-50 | 11 | 7 |
| 51+ | 4 | 7 |
| Gender |  |  |
| Female | 53 | 34 |
| Male | 26 | 20 |
| **Experience (years)ᵃ** |  |  |
| <1 | - | 1 |
| 1-5 | 25 | 17 |
| 6-10 | 30 | 15 |
| >10 | 22 | 21 |
| **Primary roleᵇ** |  |  |
| Medical - Consultant | 10 | 20 |
| Medical - Resident or Registrar | 14 | 4 |
| Nursing | 54 | 20 |
| Pharmacy | - | 4 |
| Fellow | 1 | - |
| Other^d^ | - | 6 |
| **Shocked trauma cases attended, last 3 monthsᶜ** |  |  |
| 0 | 6 | 1 |
| 1-2 | 23 | 9 |
| 3-4 | 19 | - |
| 3-5 | - | 16 |
| ≥5 or >5 | 31 | 28 |

*ᵃ Pre-implementation instrument asked years working in the emergency department; post-implementation asked years in major trauma care. Two pre-implementation responses were uncategorised.*

*ᵇ Pre-implementation instrument pooled nursing roles; post-implementation specified critical care-trained nursing (CCRN).*

*ᶜ Response categories differed between instruments and are reported as collected; cells are blank (-) where a category was not offered.*

*^d^ ‘Other’ denotes respondents who selected a primary role not among the listed options; the specific roles were not captured by the survey instrument.*

**a.	b.**

Figure S1. Standardised trauma bay set-up: (a) equipment stackers; (b) prepared trauma bay.

**Appendix S1. Shocked Trauma Care Pathway: detailed workflow, team roles, equipment standardisation, implementation strategy, and internal quality-improvement framework**

**S1.1 Pathway activation**

A patient was eligible for pathway activation when meeting standard trauma call-out criteria plus at least one of the following physiology-based triggers: systolic blood pressure ≤90 mmHg; shock index (heart rate divided by systolic blood pressure) >1; administration of any prehospital blood product; or senior clinician concern regarding early traumatic shock physiology. The prehospital blood product criterion was included because patients meeting either of the preceding physiological criteria frequently received prehospital blood and no longer met arrival thresholds. Activation triggered a 'Shocked Trauma' page, mandatory verbal notification to senior emergency, trauma and on-call consultant staff, and standardised trauma bay preparation and equipment set-up.

**S1.2 Pre-arrival processes**

Pre-arrival workflow followed a structured format: mandatory senior medical notification (emergency consultant, trauma consultant, trauma surgeon); standardised trauma bay set-up in accordance with the equipment guideline; allocation of predefined roles (trauma team leader, team leader support, airway team, haemorrhage and assessment team, scrub and procedure team, infusion and massive transfusion protocol team, radiographer, medical scribe); and a pre-arrival briefing including anticipated injuries, planned life-saving interventions, and early disposition strategy.

**S1.3 Reception and resuscitation phases**

0 to 5 minutes (immediate life-saving intervention phase): transfer to the trauma trolley in crucifix position; prioritised life-saving interventions comprising external haemorrhage control, bilateral pleural decompression for suspected tension physiology, airway obstruction management, and rapid vascular access with initiation of transfusion; truncated eFAST prioritising key views (pericardial and pleural windows); trauma series chest and pelvic radiographs; and a 5-minute team leader verbal summary.

5 to 20 minutes (completion of primary evaluation): full eFAST and secondary survey; ongoing life-saving interventions as required (for example thoracostomy or resuscitative thoracotomy); reassessment of shock physiology; and a 20-minute team leader verbal summary to establish a shared mental model.

Disposition decision: an immediate branch decision between CT and the operating theatre, determined by physiology, suspected injury burden, and response to life-saving interventions; structured handover if team leadership changed, including to the theatre team where appropriate; and a resuscitation team hot debrief initiated after disposition.

**S1.4 Team roles and human-factors design**

The trauma team configuration was task-based rather than specialty-based, aligning with human-factors principles. Roles were defined in the guideline and emphasised graded assertiveness, emotional intelligence, cognitive load management, and communication discipline.

**S1.5 Trauma bay and equipment standardisation**

The pathway required full alignment of the physical trauma bay environment, which was redesigned to ensure consistency, ease and safety of staff movement, and reduced cognitive load. This included predefined zones for team members; a decluttered environment; arm boards for vascular access; standardised invasive monitoring cables; rapid infuser (Belmont) set-up; a massive transfusion trolley mounted with a balanced transfusion whiteboard; an airway trolley with checklist and standardised medication plan; and sterile packs for thoracostomy, laparotomy, and vascular access.

**S1.6 Education, simulation and implementation strategy**

Implementation involved iterative multidisciplinary education and simulation. Translational simulation was used during development to test and refine the pathway prior to go-live. From October 2022, monthly multidisciplinary in-situ simulation was established, involving emergency, trauma, anaesthesia, intensive care, nursing and pharmacy staff, and was used both to build familiarity with equipment, roles and workflow and to return key learnings identified through ATRQI case review to the clinical floor. Additional activities included consultant-led workshops and registrar training; embedding of processes in the daily trauma team huddle; updating of institutional trauma guidelines and Emergency and Trauma Centre workflows; multidisciplinary review meetings; and continuous consultation with radiology, interventional radiology and operating theatres to streamline disposition pathways.

**S1.7 Quality improvement governance and data collection**

A quality-improvement governance structure and working group, the Alfred Trauma Resuscitation Quality Improvement (ATRQI) working group, was established in November 2022 to oversee the pathway. The group is multidisciplinary, comprising emergency, trauma surgery, anaesthesia, intensive care, radiology, nursing and pharmacy representation, and meets monthly. Activities include structured case discussion of individual shocked trauma activations; review of aggregate monthly data; hot and cold debrief structures following activation; and annual guideline revision. Monthly data review covers activation appropriateness, shock index >1 prevalence, prehospital blood administration, timeliness of life-saving interventions, balanced transfusion compliance, crystalloid volume in the emergency department, and direct-to-theatre or interventional radiology pathway utilisation.

Structured data collection was established in parallel with the clinical pathway. A dedicated trauma quality-improvement reporting form went live in October 2022 and was revised in May and November 2023. In November 2023 this process was formalised as the Alfred Trauma Resuscitation Registry, capturing prospective resuscitation-phase process data and operating alongside the established Alfred Health Trauma Registry, which captures all major trauma admissions. These indicators constitute the pathway's internal quality-improvement machinery and were not evaluation endpoints of the present report.

**Appendix S2. Pre-implementation staff survey instrument (2021)**

**Section 1. Your details**

| **Item** | **Response options** |
| --- | --- |
| Your age | 20-30 / 31-40 / 41-50 / 51+ / prefer not to specify |
| Gender | Male / Female / Non-binary / Prefer not to specify |
| Years working in ED | <1 year / 1-5 years / 6-10 years / 10+ years |
| Are you | Nursing / Medical - resident or registrar / Medical - consultant |
| How many shocked traumas have you attended in the last 3 months (not as observer) | Free text |

**Section 2. Rated items**

*All items below rated on a five-point scale: Very poor / Poor / Undecided / Good / Excellent.*

| **Domain** | **Item** |
| --- | --- |
| Trauma bay equipment (trauma bays 4-5) |  |
|  | The ease of set-up of equipment for a shocked trauma patient |
|  | The availability and accessibility of equipment during a shocked trauma patient |
|  | Ease of team-member movement to access equipment in trauma bay during resuscitation |
|  | The relevance of the equipment included in the trolleys to the needs of a shocked trauma patient |
|  | The accessibility of the patient to undertake procedures (such as insertion of lines, intercostal catheter) during a shocked trauma resuscitation, i.e. does patient positioning and equipment layout impede access |
| Free text | Is there anything you would change related to the location and availability of equipment, or from a layout perspective, in the trauma bay? If yes, please elaborate |
| Team members (based on the last three months of shocked trauma experience) |  |
|  | In-person attendance of specialty clinicians at shocked trauma call-outs, i.e. trauma surgeons, anaesthetics, when required |
|  | Completion of pre-briefing or huddle (when pre-notification received) |
|  | Team member role allocation |
|  | Team member interactions and communication |
|  | Clarity of team leadership |
|  | Team member followership |
|  | Overall team function |
| Free text | Who do you feel should attend shocked trauma call-outs? Are there barriers to them attending and if so, please elaborate |
| Time to... |  |
| Free text | Have you experienced any barriers to timeliness of life-saving interventions such as blood administration, pleural decompression, long bone splinting, management of external haemorrhage? Are there any barriers to patient transfer to disposition (CT, OT or IR)? If so, please comment |
| COVID-19 and shocked traumas |  |
|  | Availability of appropriate PPE |
|  | Appropriate PPE utilisation in the trauma bays |
|  | Location of donning area |
|  | Location of doffing areas |
|  | Use of the 'buddy system' for donning and doffing |
| Free text | How has COVID-19 changed the way you manage shocked trauma patients? What are the main challenges? |
| Free text | Are there barriers to using PPE in a shocked trauma? Please comment |
| Debriefing (based on the last three months of shocked trauma experience) |  |
|  | Colleague support during the shocked trauma |
|  | Colleague support after the shocked trauma |
|  | The post-trauma debrief |
|  | Frequency of post-trauma debrief |
|  | Follow-up after debriefing |
| Free text | Other comments on debriefing? |

**Appendix S3. Post-implementation staff experience survey instrument (2025)**

**Section 1. About you**

| **Q** | **Item** | **Response options** |
| --- | --- | --- |
| 1 | Age | 20-30 / 31-40 / 41-50 / 51+ |
| 2 | Gender | Male / Female / Non-binary / Prefer not to say |
| 3 | Years working in major trauma care | <1 year / 1-5 years / 6-10 years / >10 years |
| 4 | Your primary role | Medical - Consultant / Medical - Resident or Registrar / Nursing - CCRN / Nursing - RNG2 / Pharmacy / Other |
| 5 | Approximately how many shocked trauma cases have you attended in the last 3 months (not as an observer)? | 0 / 1-2 / 3-5 / >5 |

**Section 2. Rated items**

*Items in questions 6, 7, 9, 12, 15 and 19 rated on a five-point scale: Strongly Agree / Agree / Neutral / Disagree / Strongly disagree. Items in questions 13 and 17 additionally offered a 'Not applicable' option.*

| **Question / domain** | **Item** |
| --- | --- |
| 6. Trauma bay environment and equipment |  |
|  | Equipment required for shocked trauma resuscitation is readily available in the trauma bay and appropriate for the needs of the patient. |
|  | The trauma bay layout and equipment setup for shocked trauma patients is predictable, consistent and supports efficient movement of team members. |
|  | Patient positioning and equipment layout allow adequate access for procedures (e.g. vascular access, chest procedures). |
| 7. Team structure, leadership and communication |  |
|  | A pre-arrival team huddle or briefing occurs when pre-notification is received. |
|  | Team roles are clearly allocated prior to the resuscitation. |
|  | Leadership of shocked trauma resuscitations is clear and consistent. |
|  | Communication between team members during shocked trauma resuscitation is effective. |
|  | Team members demonstrate appropriate followership and task focus. |
|  | Senior specialty clinicians (e.g. trauma surgery, anaesthesia) attend when required. |
| 8. Free text | Who do you think should routinely attend shocked trauma call-outs? Are there any barriers to attendance? |
| 9. Timeliness and workflow |  |
|  | Life-saving interventions (e.g. blood administration, pleural decompression, haemorrhage control) are delivered in a timely manner for shocked trauma patients. |
|  | Decisions regarding patient disposition (CT vs OT or IR) are clear and well communicated. |
|  | Transfer to definitive care (OT or IR) occurs without unnecessary delay. |
| 10. Barriers experienced (tick all that apply) | Team not assembled early / Role allocation unclear / Equipment access issues / Blood product logistics / Imaging access / OT or IR access / Competing clinical demands / Communication issues / No barriers experienced |
| 11. Free text | Please describe any barriers to timely care you have experienced. |
| 12. Pathway embedding and culture |  |
|  | The shocked trauma care pathway is embedded in routine practice in our department. |
|  | The pathway promotes shared understanding of priorities during shocked trauma care. |
|  | The shocked trauma care pathway increases my confidence in my role when managing shocked trauma patients. |
| 13. Education and training |  |
|  | Education related to shocked trauma care is accessible and relevant. |
|  | In-situ simulation training has improved my familiarity with equipment, roles and workflow in caring for the shocked trauma patient. |
| 14. Free text | What education or training has been most valuable? What would you like more of? |
| 15. Debriefing and staff support |  |
|  | Colleague support during shocked trauma resuscitation is adequate. |
|  | Colleague support after shocked trauma resuscitation is adequate. |
|  | Formal debriefing occurs reliably after shocked trauma cases. |
|  | Follow-up after debriefing is appropriate when required. |
| 16. Free text | Any additional comments on debriefing or staff support? |
| 17. Shocked trauma case reviews | Questions 17 and 18 relate to the shocked trauma case review project and were not applicable to medical staff. |
|  | Shocked trauma follow-up emails were useful. |
|  | Case outcome information was helpful for further education and understanding. |
|  | Case outcome information was useful for processing and closure. |
| 18 | How many shocked trauma case reviews have you received? 0 / 1-3 / 3-5 / 5-10 / >10 / N/A |
| 19. Overall reflections |  |
|  | Overall shocked trauma resuscitation in our department is well organised and supports the provision of reliable and consistent care for this patient group. |
| 20. Free text | Please share any additional feedback or suggestions. |
